# Multiple Myeloma with Foamy Mott Cells

**DOI:** 10.1155/2021/7391895

**Published:** 2021-08-10

**Authors:** Karen Nalbandyan, Daniel Benharroch, Benzion Samueli, Michael Kafka, Anna Gourevitch

**Affiliations:** ^1^Department of Pathology, Soroka University Medical Center, Faculty of Health Sciences, Ben-Gurion University of the Negev, Beer-Sheva, Israel; ^2^Division of Hematology, Hematology Laboratory, Soroka University Medical Center, Faculty of Health Sciences, Ben Gurion University of the Negev, Beer-Sheva, Israel; ^3^Division of Hematology, Soroka University Medical Center, Faculty of Health Sciences, Ben-Gurion University of the Negev, Beer-Sheva, Israel

## Abstract

Intracytoplasmic assorted vacuoles containing immunoglobulin collections are occasionally seen in multiple myeloma. When abundant, they impart a foamy appearance to the tumor cells, which is a potential source for diagnostic pitfalls. Herein, we report the case of a patient who presented with skeletal pain and CT confirmed lytic lesions. A bone marrow biopsy revealed multiple myeloma with unusual foamy Mott cells. The patient was subsequently treated with four cycles of cyclophosphamide, bortezomib, and dexamethasone induction therapy, followed by 3 cycles of lenalidomide with dexamethasone. A biopsy performed following initial biological and immunomodulatory drugs revealed different morphological and clonal characteristics. These features were modified again, five years later, and again, after two years of close monitoring. Hematopathologists should be aware of this morphologic variant of myeloma as well as for the capacity of clonal characteristics, such as light chain monotype, to fluctuate subsequent to treatment.

## 1. Introduction

Multiple myeloma is a multifocal plasma cell neoplasm associated with an M-protein in the serum and/or urine [1]. Myeloma cells show a wide spectrum of morphological variation, from a near normal plasmacytic phenotype to pleomorphic multinucleated or anaplastic forms. As a rule, the tumor cells are larger than their nonneoplastic counterparts; however, normal and small cell sized variants have been described. Other variations include nuclear convolutions and Burkitt-like or blastic morphology [2]. Occasionally, intracellular immunoglobulin (Ig) accumulations are seen within variable sized vacuoles. Such inclusions are called Dutcher bodies if seemingly intranuclear and Russel bodies if intracytoplasmatic; when numerous, the cell carrying them is termed a Mott cell. Here, we report a rare case of multiple myeloma with foamy cell features. The finding may be significant as a potential source of diagnostic pitfall.

## 2. Case Presentation

### 2.1. Clinical Summary

A 65-year-old Caucasian male presented in May 2015 for evaluation of right-shoulder and upper-back pain for the preceding 6 months. Computed tomography (CT) showed a paraspinal soft tissue mass, compression of the D7 and D8 vertebrae, and lytic lesions in the right scapula and D9 vertebra. Blood tests revealed the following: hemoglobin 13.6 g/dl, creatinine 1.45 mg/dl, globulins 5.9 g/dl, with a monoclonal peak of IgG kappa of 3.9 g, free kappa light chain 165 mg/dl, and kappa/lambda ratio 22.10. Urine protein was 5.7 g/l, and Bence-Jones protein was negative. Bone marrow investigation was performed in relation with a working diagnosis of multiple myeloma.

### 2.2. Pathological Findings

Bone marrow biopsies obtained from the patient were fixed in 4% buffered formaldehyde (Gadot Biochemical Industries, Haifa, Israel), decalcified in 10% EDTA decalcification solution (Milestone, Italy), processed at 40°C, and embedded in paraffin blocks at 61°C in Tissue-Tek VIP6-E2 processor (Sakura), according to the standard protocol of the laboratory. Sections of 5–10 *μ*m in thickness were stained with Mayer's hematoxylin (Pioneer/Sigma) and eosin (Pioneer Research Chemicals, Ltd.) in Tissue-Tek Prisma E-2S automated slide stainer (Sakura). The immunostainings and chromogenic in situ hybridization (CISH) were carried out on a BenchMark XT Full System (Ventana, Tucson, AZ, USA) using antibodies to the following: CD68 clone PG-M (Dako), anti-kappa (clone L1C1) and anti-lambda (clone Lamb14), both from CellMarque, and anti-cytokeratin antibody clones AE1/AE3 and MNF116. Cytoplasmic kappa and lambda mRNA CISH probes were ready to use (Ventana). Histological examination was performed using an Olympus BX41 microscope (Olympus, Tokyo, Japan). Smears from bone marrow aspirates were prepared manually and stained with Wright stain by an automatic Sysmex SP50 slide stainer.

On bone marrow aspiration, cells of unequivocal plasmacytic morphology comprised 4% of all leukocytes. In addition, 20% of total leukocytes were represented by very large cells with eccentric nuclei and highly vacuolated cytoplasm. Their differential diagnosis included Mott cells and macrophages.

**Figure 1:**
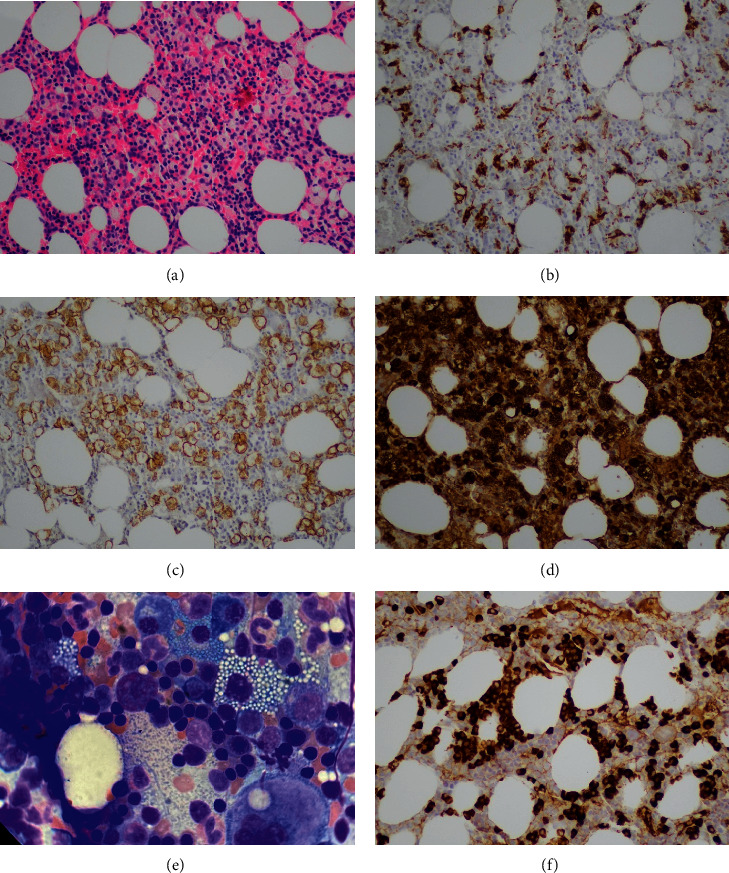
Bone marrow biopsy with foamy Mott cells on (a) H&E immunostain for (b) CD68 highlights scattered smaller cells of macrophage-monocyte lineage, but are negative in the larger foamy cells; however, they are positive for (c) CD138 and (d) kappa, confirming their plasmacytic differentiation. Note that the cytoplasmatic kappa IHC pattern matches the inclusions seen on H&E. (e) Bone marrow aspirate smear (Wright stain). (f) Posttherapy bone marrow biopsy, lambda light chain. Trephine biopsy cells original magnification 200x; aspirate smear (cell (E)) original magnification 1000x.

The bone marrow biopsy was mildly hypercellular for age (about 75%), predominantly erythroid lineage. Numerous large cells with foamy cytoplasm, resembling foamy macrophages or Gaucher cells (Figure 1(a)), were identified. The large cells stained negative for CD68 PGM1 (Figure 1(b)), periodic acid-Schiff (PAS), and both cytokeratin AE1/AE3 and MNF116. They showed strong positive cellular membrane staining for CD138 (Figure 1(c)); the staining for kappa light chain was positive, cytoplasmatic, in 60% of the marrow nucleated cells (Figure 1(d)), whereas lambda light chain was positive in isolated cells only (<1%). A FISH confirmed kappa light chain restriction, consistent with monoclonality. The patient was subsequently treated with 4 cycles of cyclophosphamide, bortezomib, and dexamethasone induction therapy, followed by 3 cycles of lenalidomide with dexamethasone (Rd), which was marked by a good partial response.

On follow-up, bone marrow biopsy was performed in December 2015 (7 months after initial presentation), the large vacuolated cells comprised only 5% of the cellular marrow (Figure 1(e)). However, the marrow showed prominent (50%) infiltration by medium sized atypical plasma cells, without vacuolization, highlighted by CD138. This population was positive for lambda light chain (Figure 1(f)). Serum immunofixation revealed a new IgG with lambda in addition to the original IgG with kappa light chains. Repeat immunofixations consistently demonstrated this biclonality. An electron microscopy study was attempted on a follow-up aspiration specimen, but it was not successful. The patient was maintained on the same treatment with a very good partial response.

In June 2016, the patient received high dose melphalan and an autologous bone marrow transplantation. The patient was treated with lenalidomide maintenance for 2 years. The patient maintained a stable partial response during that time, which lasted until January 2020. No progression was noted per serum protein electrophoresis or by repeated bone marrow examinations. In December 2020 (5 years after initial treatment), a serum protein electrophoresis and immunofixation (SPEP) disclosed an M spike which was 0.6 g/dL, IgG 1992 g/dL, kappa light chain 1825 g/dL, and free light chain ratio (FLCR) of 3.03. PET CT showed older, multiple lytic bone lesions without FDG uptake. A positive immunofixation with an elevated bone marrow monotypic kappa light chain are considered to represent progression of disease. Bone marrow biopsy taken at this time showed an interstitial infiltration comprising about 30% of the bone marrow, composed of large cells, displaying both morphology and phenotype (including kappa restriction) identical to those seen in the original biopsy (2015). In our opinion, this represented a reemergence of the initial tumor clone. At this point, continued close monitoring was applied.

In January 2021, the patient's disease was reevaluated. BMB revealed a 40% infiltration of kappa light chain monotypic plasma cells, with widespread large Mott cells. SPEP showed M spike of 0.64 g/dL, IgG 2085 g/dL, and kappa light chain 1979 g/dL with FLCR 3.97. The patient's CBC and chemistry were normal. A plan has been initiated to treat the patient with carfilzomib and daratumumab with dexamethasone.

## 3. Discussion

We present a case of multiple myeloma with “foamy” myeloma cells. A few conditions with similar morphology have been described in multiple myeloma. The cytoplasm of typical Mott-type plasma cells contains globules composed of retained immunoglobulins [3, 4]. However, classically the vacuoles are larger than in the present case and the residual cytoplasm is basophilic; therefore, they usually do not present a diagnostic difficulty. In contrast, large cells with a foamy cytoplasm, as in the present case, should raise suspicion of Gaucher cells. However, negative stains for CD68 PGM1 are not in favor of Gaucher cells. On the other hand, infrequently so-called Gaucher-like or pseudo-Gaucher cells have been described in multiple myeloma, corresponding to macrophages, filled with variable contents [5]. The large cells described in our case are morphologically reminiscent of these Gaucher-like cells. However, pseudo-Gaucher cells are of histiocytic lineage [6, 7], as should be confirmed by their positivity for CD68, while in our case the foamy cells were exclusively plasmacytes, confirmed by an immunophenotype which was negative for CD68 and positive for CD138 and kappa light chain. A population of large plasma cells with numerous crystalline intracytoplasmic inclusions has also been described in crystal-storing histiocytosis [8]. However, such large plasma cells are admixed with bona fide foamy histiocytes, which was not the case for our patient. In our opinion, the large cells seen in the present case were most probably a variant of Mott cells harboring numerous unusually small intracytoplasmic immunoglobulin globules. Due to this feature, the cytoplasm of Mott cells appears abundant and foamy, mimicking histiocytes seen in some storage diseases.

The findings in the follow-up biopsies seem to reflect concomitant involvement by two tumor clones of different cytological and immunohistochemical phenotypes. The initially diagnosed clone was comprised of the morphologically foamy Mott cells and was kappa light chain restricted. This clone showed partial response to treatment, at which point a second lambda light chain restricted clone emerged, which was refractory to treatment. Such an evolution probably represents an example of the so-called clonal competition with alternating dominance, previously described and discussed [9], wherein under various therapy regimens, some tumor clones are suppressed while others take proliferative advantage, becoming more dominant. This phenomenon may also explain the relapse of the kappa light chain positive, foamy Mott cell clone in 2020, confirmed and expanded in June 2021.

Multiple myelomas with predominantly mature plasma cell phenotype (so-called Marschalko subtype) show a significantly better prognosis than immature variants (e.g., plasmablastic myeloma) [5]. Nevertheless, current prognostic schemes for multiple myeloma do not include a morphological classification of the tumor [5].

In conclusion, we have described a case of multiple myeloma with the unusual morphology of the Mott cells. The unique foamy appearance was imparted by small intracytoplasmic immunoglobulin globules. These cells closely simulate foamy macrophages or Gaucher cells and may be the source of a diagnostic error. Appropriate immunohistochemistry, including CD68, CD138, kappa, and lambda, along with clinical correlation, easily resolves the diagnosis. A follow-up biopsy consequent to therapy showed a near complete response of the original tumor with emergence of a second myeloma clone with different cytological and immunohistochemical phenotype and then subsequent reemergence of the initial, foamy Mott cell clone after five years of follow-up. Such changes may represent clonal competition with alternating dominance.

## Abbreviations

Ig: Immunoglobulin
EDTA: Ethylenediaminetetraacetic acid
CISH: Chromogenic in situ hybridization
CT: Computed tomography
PAS: Periodic acid-Schiff
H&E: Hematoxylin and eosin stain
IHC: Immunohistochemistry.
